# Artificial Intelligence and Colposcopy: Detection and Classification of Vulvar HPV-Related Low-Grade and High-Grade Squamous Intraepithelial Lesions

**DOI:** 10.3390/jcm14197065

**Published:** 2025-10-07

**Authors:** Miguel Mascarenhas, Vanitha Sivalingam, Inês Castro, Katie Jones, Miguel Martins, Inês Alencoão, Maria João Carinhas, Joana Mota, Pedro Cardoso, Francisco Mendes, Maria João Almeida, Bruno Mendes, João Ferreira, Guilherme Macedo, Teresa Mascarenhas, Ahsan Javed, Rosa Zulmira Macedo

**Affiliations:** 1 Precision Medicine Unit, Department of Gastroenterology, São João University Hospital, 4200-427 Porto, Portugal; miguelpedro96@gmail.com (M.M.); francisco.cnm@gmail.com (F.M.); maj.almeida.14@gmail.com (M.J.A.);; 2 Faculty of Medicine, University of Porto, 4200-135 Porto, Portugal; 3 Department of Gynecology, Liverpool Women’s NHS Foundation Trust, Liverpool L8 7SS, UK; 4 Department of Gynecology, Centro Materno-Infantil do Norte Dr. Albino Aroso (CMIN), Santo António University Hospital, 4050-651 Porto, Portugalinesalencoao@gmail.com (I.A.);; 5 Department of General Surgery, Liverpool University Hospital NHS Foundation Trust Liverpool, Liverpool L7 8YE, UK; katie.jones@liverpool.ac.uk (K.J.); ahsanj@liverpool.ac.uk (A.J.); 6 Faculty of Engineering, University of Porto, 4200-072 Porto, Portugaljferreiraa@fe.up.pt (J.F.); 7 Department of Gynecology, São João University Hospital, 4200-135 Porto, Portugal

**Keywords:** vulvar squamous cell carcinoma, HSIL, LSIL, colposcopy, artificial intelligence

## Abstract

**Background/Objectives**: Accurate identification of vulvar high-grade squamous intraepithelial lesions (HSIL) is essential for preventing progression to invasive squamous cell carcinoma. This study addresses the gap in artificial intelligence (AI) applications for vulvar lesion diagnosis by developing and validating the first convolutional neural network (CNN) model to automatically detect and classify HPV-related vulvar lesions—specifically HSIL and low-grade squamous intraepithelial lesions (LSIL)—based on vulvoscopy images. **Methods**: This bicentric study included data from 28 vulvoscopies, comprising a total of 9857 annotated frames, categorized using histopathological reports (HSIL or LSIL). The dataset was divided into training, validation, and testing sets for development and assessment of a YOLOv11-based object detection model. **Results**: The CNN demonstrated a recall (sensitivity) of 99.7% and a precision (positive predictive value) of 99.1% for lesion detection and classification. **Conclusions**: This is the first AI model developed for detecting and classifying HPV-related vulvar lesions. The integration of such models into vulvoscopy could significantly improve diagnostic accuracy and positively impact women’s health by reducing the need for potentially invasive and anatomy-altering procedures.

## 1. Introduction

Vulvar cancer is relatively uncommon, accounting for 4% of all gynecologic cancers [1], with approximately 47,336 new cases diagnosed worldwide in 2022 [2]. Squamous cell carcinoma (SCC) is the predominant histologic subtype, representing over 90% of malignant vulvar tumors, most of which originate from vulvar intraepithelial lesions (VIN), often associated with human papillomavirus (HPV) infection [3].

Two distinct carcinogenic pathways have been identified: one HPV-related, involving high-grade squamous intraepithelial lesion (HSIL, or VIN usual type) and another HPV-independent, associated with chronic inflammatory conditions, such as lichen sclerosus, leading to differentiated VIN (dVIN) [3,4]. These differing etiologies result in distinct clinical and pathological profiles.

Anatomically, the vulva consists of keratinized squamous epithelium and lacks a transformation zone, influencing how HPV infection affects this region compared to the cervix and anus [5]. According to the 2015 classification of the International Society of Study of Vulvovaginal Diseases (ISSVD), low-grade squamous intraepithelial lesion (LSIL, previously referred to as VIN1) includes benign HPV-related skin changes, such as flat condyloma, and it is not considered a precancerous lesion [5,6].

In contrast, vulvar HSIL represents a true precancerous lesion and is associated with high-risk HPV types, particularly HPV 16, 18, and 45. These lesions typically present as multifocal and are often found around the introitus and labia minora, and affect predominantly younger women (median age: 48 years). The estimated 10-year risk of progression to invasive cancer is 10%. Differentiated VIN (dVIN), by contrast, is less prevalent, occurs more frequently in postmenopausal women (median age: 60 years), and carries a substantially higher risk of malignant transformation with a 10-year cancer risk progression up to 50% [4,7,8,9]. Current consensus recommends reserving the term “VIN” exclusively for vulvar HSIL [5,6].

Despite the well-established role of HPV in vulvar carcinogenesis, there are no screening strategies for the early detection of vulvar HSIL [10]. Vulvar precancerous lesions may present with pruritus (60% of cases) but are often asymptomatic [5]. Most lesions are visible on physical examination, and visualization can be improved using magnification tools such as colposcopes. Vulvoscopy, as a component of genital tract examination during colposcopy, enables a systematic examination of the vulva, and the use of 5% acetic acid can improve lesion detection [11]. There are no colposcopic pathognomonic markers for vulvar lesions. HSIL typically presents as multifocal, discoid lesions that are white or pigmented, often raised, and may extend to the perianal skin. These are frequently associated with other HPV-related lesions of the genital tract. LSIL may appear as condylomatous growths or as subtle cutaneous alterations, which can be pigmented or non-pigmented and either flat or elevated. dVIN generally manifests as small, solitary white or red lesions, developing in the background of dermatological and anatomical changes related to chronic inflammation, such as lichen sclerosus [12]. Although aceto-whitening has high sensitivity (97%), its specificity is low (40%) as a predictor of HSIL [13], and dVIN typically does not react to acetic acid [4]. Therefore, even with the aid of staining, vulvoscopy may yield suboptimal diagnostic accuracy and is subject to considerable intra- and interobserver variability, particularly when multiple or morphologically diverse lesions from multiple carcinogenesis pathways can be present. Diagnosis relies more heavily on visual inspection and histopathological confirmation than on cervical or vaginal evaluation, given the absence of highly specific visual features [12].

Given these challenges in lesion recognition and classification during vulvoscopy, artificial intelligence (AI) holds promise as a tool to enhance its diagnostic precision and reproducibility. Convolutional Neural Networks (CNNs), a class of deep learning models, have achieved high accuracy in cervical and vaginal lesion classification [14,15]. Colposcopic devices can, in fact, assess the entire genital tract, including the cervix and vaginal walls, but also the vulva areas. However, to our knowledge, no published studies have specifically explored the use of AI algorithms for classifying vulvar lesions.

In this first study, we focused on HPV-related vulvar lesions, due to their higher prevalence and clearer diagnostic profile, reserving dVIN for future research. The aim was to develop and validate a CNN to detect and classify HPV-related vulvar lesions—specifically HSIL and LSIL—based on vulvoscopy images.

## 2. Materials and Methods

### 2.1. Dataset Collection and Annotation

For this study, vulvoscopies were retrospectively obtained from two institutions between February 2023 and February 2025: Centro Materno Infantil do Norte (Porto, Portugal) and Liverpool Women’s NHS Foundation (Liverpool, UK). These procedures were performed by one expert at each location, consistent with established best practices. The procedures were recorded using Carl Zeiss FC-150 (Carl Zeiss Vision GmbH, Aalen, Germany) and Seiler 955 5Step colposcopes (Seiler Medical, Denver, CO, USA), respectively, and conducted by dedicated experts, ensuring compliance with current best practices.

We developed a deep learning model designed to automatically detect and classify vulvar lesions as either LSIL or HSIL. The histological report from the biopsy obtained during the procedure was considered the gold standard reference. The dataset exclusively included images from biopsied vulvar lesions. Each frame containing a biopsied lesion was annotated with a bounding box by consensus between two expert physicians; only agreed-upon annotations were included. In total, 9857 annotated frames containing biopsy-confirmed lesions, comprising 6749 LSIL and 3108 HSIL frames/objects (each frame had one object annotated). We included frames encompassing all stages of vulvoscopy, including non-stained, stained with acetic acid, and after a lesion biopsy/treatment.

### 2.2. Dataset Preparation and Model Development

**Figure 1:**
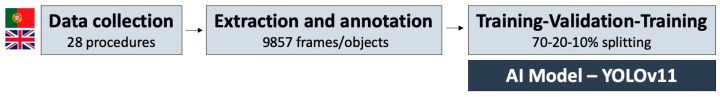
Design of the study.

We split the dataset into three parts: a training set (*n* = 6899 frames, 70%), a validation set for tracking model performance (*n* = 1972 frames, 20%), and a test set (*n* = 986 frames, 10%) in which the model was evaluated with a threshold for confidence score that maximized the F1-score of the validation set. No patient split was performed to maximize data use. Figure 1 summarizes the design of the study.

**Figure 2:**
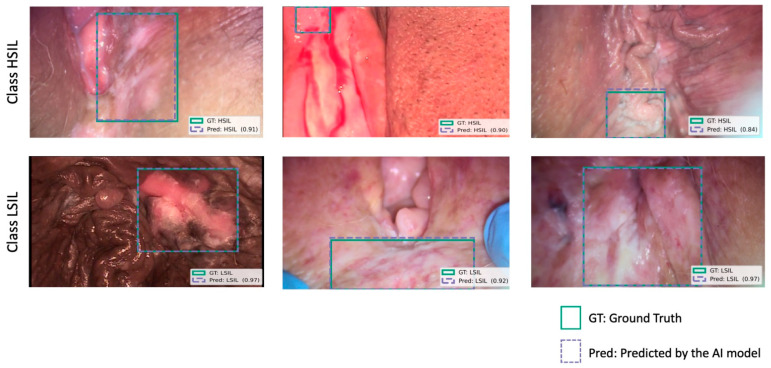
Examples of correct model predictions for vulvar HSIL (**first row**) and LSIL (**second row**) lesions. Blue boxes indicate predicted bounding box generated by the model. Green boxes represent ground truth annotated by consensus of expert physicians. The model’s confidence score for each prediction is also presented in parentheses, indicating the certainty of the classification. Images were selected to illustrate variability in lesion appearance, lighting conditions, different devices, and procedural stage (including non-stained, acetic-acid-stained, and post-biopsy frames).

We used a YOLOv11-based object detection model to detect and classify vulvar lesions (detect: locating the area of the image with the highest probability of the lesion being present, marking it with a bounding box; classify: assigning the detected lesion one of the two possible categories—“LSIL” or “HSIL”). The model processed input images to identify regions containing lesions by generating bounding boxes around the region of interest. Each bounding box was then associated with a specific diagnostic class, in this case as “LSIL” or “HSIL”, based on visual features learned during training, allowing for simultaneous localization and classification of lesions (Figure 2). To achieve this, the model first processed input data using a deep neural network to extract a high-level feature map from the input image. This map was divided into an N × N grid, where each grid cell was responsible for detecting objects whose centers fell primarily within its specific spatial boundary.

The model was trained end-to-end, with all layers updated during the optimization process. Training was performed for up to 50 epochs, with early stopping applied based on the validation loss, using a patience threshold of 10 epochs to prevent overfitting. The total loss function was composed of three elements: a box regression loss (mean squared error), a classification loss (cross-entropy), and a distribution focal loss, reflecting the multi-task nature of lesion detection and classification.

### 2.3. Post-Processing and Model Performance Assessment

We applied non-maximum suppression (NMS) to refine the detected bounding boxes by applying the Intersection over Union (IoU) metric between predicted and annotated boxes, along with a confidence score to retain the most confident bounding box. We carefully selected the NMS confidence and IoU thresholds to optimize the YOLOv11 model’s performance, balancing precision and recall in lesion identification.

Model performance was evaluated on the test set, with object-detection metrics. The primary outcomes were recall (the same as sensitivity), precision (the same as positive predictive value), mean average precision at IoU ≥ 0.50 (mAP50), and mean average precision across IoU thresholds from 0.50 to 0.95 (mAP50-95). The statistical analysis was performed using Sci-kit learn version 0.22.2.

### 2.4. Ethical Considerations

This non-interventional study adhered to the Declaration of Helsinki’s ethical principles and received approval from the ethical committee (IRB 2023.157 (131-DEFI/123-CE)). To ensure data privacy, all potentially identifying information was removed, and each patient’s data was assigned a random, anonymized number before being incorporated into the dataset. A certified legal team with data protection expertise further guaranteed that the data remained untraceable, fully complying with General Data Protection Regulation (GDPR) standards.

## 3. Results

**Figure 3:**
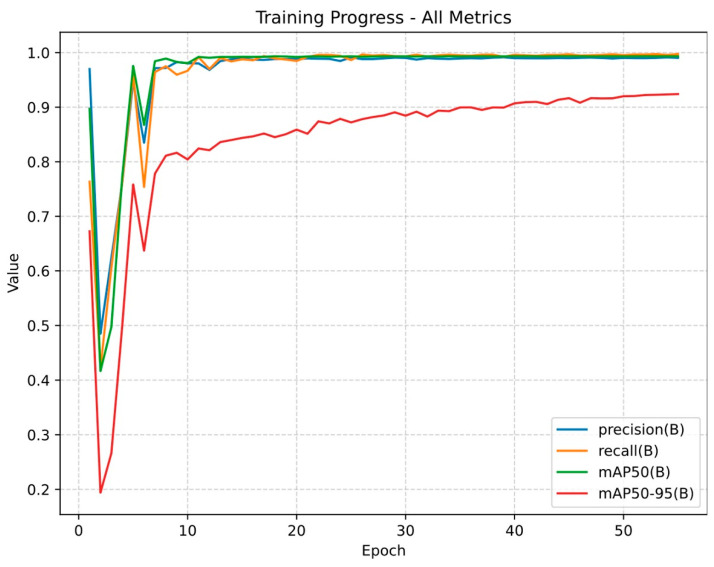
Training progress of the YOLOv11-based model across 55 epochs. The y-axis represents the normalized performance metrics (ranging from 0 to 1), and the x-axis represents the number of training epochs. In this context, an epoch refers to one complete iteration over the training dataset during model development. Precision (the same as positive predictive value) is the proportion of the predicted lesions that are actually correct. Recall (the same as sensitivity) is the proportion of true lesions that the model detected. mAP50 (mean average precision at 50% Intersection over Union, IoU) represents the percentage of predicted bounding boxes that overlap with the ground-truth annotation by at least 50%. mAP50–95 extends this metric by averaging performance across a stricter range of IoU thresholds (from 0.50 to 0.95).

The dataset included 9857 annotated frames containing biopsy-confirmed lesions, from 28 vulvoscopies (9 from Centro Materno Infantil do Norte, 19 from Liverpool Women’s NHS Foundation). Figure 3 schematizes the evaluation of main outcomes through the training and validation process.

When evaluated on test set to assess the classification performance, the model demonstrated a recall/sensitivity of 99.7% (the proportion of true lesions that the model detected), indicating its ability to detect nearly all lesions, while its precision/positive predictive value was 99.1% (the proportion of the predicted lesions that are actually correct), reflecting a low rate of false-positive detection.

When evaluated on a test set to assess the localization performance, the model achieved a mean precision of 99.3% at an IoU threshold of 0.50 (mAP50), indicating the proportion of predicted bounding boxes that overlapped with the ground-truth annotation by at least 50%. Under a stricter range of IoU threshold from 0.50 to 0.95, the model demonstrated a mean precision of 92.4% (mAP50-95).

**Figure 4:**
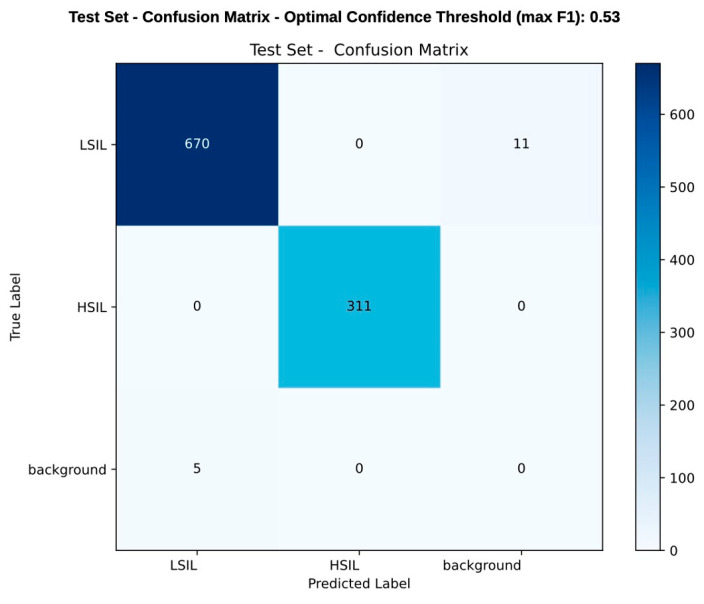
Frame-level confusion matrix comparing the model’s prediction with histopathological classification (considered the ground truth). Each value represents the number of frames in which a lesion was either correctly or incorrectly detected and classified. A frame is considered correctly classified if it contains a detected lesion matching the true label. During the test set, the model correctly predicted 670 LSIL cases and 311 HSIL cases, while 11 LSIL cases were misclassified as background, and 5 background areas were mistakenly labeled as LSIL. The scale on the right side of the table represents the color gradient corresponding to the frequency of predictions, with darker shades indicating higher values. This matrix reflects detection and classification performance rather than a conventional diagnostic accuracy/confusion matrix. As the YOLOv11 model detects and classifies lesions simultaneously, metrics like specificity and negative predictive value were not applicable due to the limited number of background frames.

Figure 4 provides a frame-level confusion matrix, where a frame is considered a true positive if it includes a correctly detected lesion. However, this table should not be interpreted as a conventional confusion matrix comprising true positives, true negatives, false positives, and false negatives. Due to the nature of the AI model used (YOLOv11-based object detection model), the performance assessment was focused on the model’s ability to simultaneously detect and differentiate lesions. Given the low number of background frames, conventional metrics as specificity and negative predictive value could not be reliably calculated.

## 4. Discussion

Detecting vulvar intraepithelial lesions is challenging, even for experienced gynecologists and dermatologists. To our knowledge, this is the first proof-of-concept AI developed for detecting and classifying HPV-related vulvar lesions. The AI model demonstrates high performance metrics, effectively detecting and distinguishing HSILs and LSILs within vulvar frames with both recall and precision of 99%. This advancement could significantly improve the detection rates of clinically important lesions, signaling the need for biopsy and reducing the number of vulvar cancers that are missed due to delayed biopsies [11].

In fact, vulvoscopy is a procedure requiring specialized training and expertise due to its high false-positive rate and the difficulty in distinguishing vulvar lesions based on macroscopic appearance [4]. Therefore, recognizing lesions that require biopsy and treatment, as opposed to benign or physiological changes, is of the highest importance. Deep learning models are becoming indispensable tools in image-based specialties for diagnostic and therapeutic evaluation. While other AI models for vulvar pathology are being developed, they primarily focus on benign conditions such as venereal dermatoses and lichen sclerosus [16,17]. AI models for the gynecological assessment of HPV-related lesions remain in their early stages and are mainly limited to cervical and vaginal applications [18]. However, vulvar precancerous lesions significantly differ from their cervical and vaginal counterparts. Vulvar LSIL, which includes flat condyloma and HPV-related skin changes, is commonly associated with low-risk HPV strains like HPV 6 or 11 and is not considered precancerous [3,4]. In contrast, vulvar HSIL is the precursor to HPV-related invasive carcinoma, with oncogenesis comparable to cervical, vaginal, and anal HSIL. Notably, up to 60% of vulvar cancers are HPV-negative, whereas only 10% of vulvar pre-invasive lesions are differentiated VIN [19].

This AI model marks a significant milestone as the first dedicated AI system for detecting HPV-related vulvar lesions. The model was trained using histologic biopsy results from specific vulvar sites (ground truth), and it functions as both a CADe (computer-aided detection) and CADx (computer-aided diagnosis) system by detecting lesions through an explainable AI mechanism (bounding box) and classifying them as HSIL or LSIL. This increases the technological readiness level of such a model, as it has the potential to enhance the diagnosis performance for both expert and young physicians in a trustworthy manner. This is especially important considering the black box nature of deep learning models.

Additional strengths of this study include the incorporation of still frames from the entire procedure, encompassing non-stained, stained, and manipulated frames (via biopsy or laser treatment). This comprehensive dataset exposed the model to a diverse range of clinically relevant visual information, including tissue alterations and blood presence. Furthermore, the model was trained on HPV-related lesions located in various vulvar regions, viewed from different angles and magnifications. This diverse exposure enhances the model’s clinical utility by improving its adaptability to real-world settings.

The development of such a vulvar AI model could pave the way for software as a medical device that would be highly useful in clinical settings and help reduce both intra- and interobserver variability between physicians [20]. Compliance with the FAIR principles, which state that the AI models should be Findable, Accessible, Interoperable, and Reusable [21]. By incorporating data from two different types of colposcopes, we enhance the model’s interoperability and extend its clinical value.

However, certain methodological limitations should be acknowledged when interpreting these results. This study is retrospective and was conducted at two international centers with a relatively small dataset. As a result, demographic bias (selection bias) cannot be ruled out, potentially affecting the external validity of the findings and suggesting that performance accuracy may vary in broader clinical settings. Additionally, the potential data leakage secondary to the lack of a procedural-level split between sets could potentially contribute to the overestimation of performance metrics. To address this limitation, we closely monitored model performance on validation and test sets to ensure a robust evaluation. Moreover, each frame included only one lesion, and only HPV-related precancerous vulvar lesions were considered. Rare but aggressive dVIN, as well as other lesions such as melanoma and vulvar Paget disease, were also not included. Nonetheless, we believe this study remains valuable to share due to its uniqueness as the first developed model for HPV-related vulvar lesions detection and classification.

While potentially impactful, the model’s performance in real-life video scenarios remains uncertain, and prospective multicentric studies are still needed. Considering the heterogeneity of vulvar lesions, we should also strive to develop a more robust and diversified dataset, including data from additional centers, that can further contribute to answering other clinical problems that committed physicians frequently encounter.

## 5. Conclusions

This study represents a crucial step toward AI integration in vulvar lesion detection and differentiation. Given vulvoscopy’s low sensitivity in detecting vulvar HSIL, integrating AI-enhanced vulvoscopy could substantially improve diagnostic accuracy and positively impact women’s health by reducing the need for potentially invasive and anatomy-altering procedures. This study is noteworthy because it is the first to be able to distinguish HPV-related HSIL from LSIL, but also because it employs a YOLO model that performs detection and classification simultaneously.

Colposcopy enables ubiquitous assessment of the cervix, vagina, and vulva. However, current AI publications are mainly focused on the cervical and vaginal evaluations. By extending AI models to vulvar assessment, as demonstrated in this first study, we move closer to achieving a ubiquitous AI-enhanced colposcopic assessment of the female genital tract.

## Data Availability

The data are available upon reasonable request.
